# Polymorphisms at the *F12* and *KLKB1* loci have significant trait association with activation of the renin-angiotensin system

**DOI:** 10.1186/s12881-016-0283-5

**Published:** 2016-03-11

**Authors:** Nilima Biswas, Adam X. Maihofer, Saiful Anam Mir, Fangwen Rao, Kuixing Zhang, Srikrishna Khandrika, Manjula Mahata, Ryan S. Friese, C. Makena Hightower, Sushil K. Mahata, Dewleen G. Baker, Caroline M. Nievergelt, Sucheta M. Vaingankar, Daniel T. O’Connor

**Affiliations:** Departments of Medicine, University of California at San Diego, 9500 Gilman Drive, La Jolla, 92093-0838 USA; Veteran Affairs (VA) San Diego Health Care System and VA Center of Excellence for Stress and Mental Health, 3350 La Jolla Village Drive, San Diego, 92161 USA; Department of Psychiatry, University of California at San Diego, 9500 Gilman Drive, La Jolla, 92093-0838 USA; Departments of Pharmacology, Institute of Genomic Medicine, University of California at San Diego, 9500 Gilman Drive, La Jolla, 92093-0838 USA

**Keywords:** FXIIa (active protease encoded by gene *F12* or Hageman factor), Kallikrein/KAL (active protease encoded for by gene *KLKB1* or Fletcher factor), rs3733402, rs1801020, PTSD (posttraumatic stress disorder), Hypertension

## Abstract

**Background:**

Plasma coagulation Factor XIIa (Hageman factor; encoded by *F12*) and kallikrein (KAL or Fletcher factor; encoded by *KLKB1*) are proteases of the kallikerin-kinin system involved in converting the inactive circulating prorenin to renin. Renin is a key enzyme in the formation of angiotensin II, which regulates blood pressure, fluid and electrolyte balance and is a biomarker for cardiovascular, metabolic and renal function. The renin-angiotensin system is implicated in extinction learning in posttraumatic stress disorder.

**Methods & Results:**

Active plasma renin was measured from two independent cohorts- civilian twins and siblings, as well as U.S. Marines, for a total of 1,180 subjects. Genotyping these subjects revealed that the carriers of the minor alleles at the two loci- *F12* and *KLKB1* had a significant association with reduced levels of active plasma renin. Meta-analyses confirmed the association across cohorts. In vitro studies verified digestion of human recombinant pro-renin by kallikrein (KAL) to generate active renin. Subsequently, the active renin was able to digest the synthetic substrate angiotensinogen to angiotensin-I. Examination of mouse juxtaglomerular cell line and mouse kidney sections showed co-localization of KAL with renin. Expression of either *REN* or *KLKB1* was regulated in cell line and rodent models of hypertension in response to oxidative stress, interleukin or arterial blood pressure changes.

**Conclusions:**

The functional variants of *KLKB1* (rs3733402) and *F12* (rs1801020) disrupted the cascade of enzymatic events, resulting in diminished formation of active renin. Using genetic, cellular and molecular approaches we found that conversion of zymogen prorenin to renin was influenced by these polymorphisms. The study suggests that the variant version of protease factor XIIa due to the amino acid substitution had reduced ability to activate prekallikrein to KAL. As a result KAL has reduced efficacy in converting prorenin to renin and this step of the pathway leading to activation of renin affords a potential therapeutic target.

## Background

Hypertension is a global public health issue and contributes to the burden of heart disease, stroke, kidney failure and premature mortality (13 % of total deaths worldwide)[1]. The kidney serves as a major organ for maintaining normal blood pressure (BP) and the local renal renin angiotensin system (RAS) pathway acts as the master regulator of renal function during hypertension [2–4]. The renin-angiotensin-aldosterone system (RAAS) is a signaling pathway responsible for regulating the body's blood pressure [5–8]. Stimulated by low BP the kidney releases renin, this triggers a signal transduction pathway generating eventually angiotensin II that causes vasoconstriction, leading to increase in BP. Several cardiovascular therapies for high BP, target the RAAS system and these therapies are now being explored for their efficacy in treating PTSD [9, 10].

The juxtaglomerular (JG) cells in the kidney express renin a member of the aspartyl protease family. It is the limiting enzyme in RAS pathway that converts angiotensinogen to angiotensin I (Ang I) [11]. Renin production is tightly regulated at the transcriptional level and the active renin is released into the circulation through regulated exocytosis [11, 12]. About 80 % of the renin present in plasma is in an enzymatically inactive form called pro-renin. Kidney processes inactive pro-renin to renin and is the major source of circulating active renin in humans. The plasma renin concentration contributes significantly to cardiovascular and renal diseases like hypertension, coronary heart disease, and chronic kidney disease [13]. Thus the conversion of pro-renin to renin is a potential regulatory site for therapeutic intervention.

We studied the effect of the *KLKB1* (located on chromosome 4) missense variant rs3733402 (Asn124Ser) on circulating levels of active renin and observed that homozygous carriers of the minor allele (Ser/Ser) displayed lower levels of active renin [14]. In vitro proteolysis and cell biology indicated that pro-renin was a substrate for plasma kallikrein (KAL). The KAL-activated renin in turn, was able to cleave substrate angiotensinogen to angiotensin 1 the precursor for vasoconstrictor angiotensin II. Situated on chromosome 5, the coagulation factor *F12* 5’-UTR variant rs1801020 also showed significant association with plasma levels of active renin. The *F12* locus encodes for the FXIIa protease responsible for converting pre-kallikrein to KAL. The possible implication of the intrinsic coagulation system and the fibrinolytic system in renin activation has been discussed. In both the independent cohorts a strong association was observed between levels of active renin and occurrence of the minor alleles.

## Methods

### Twin and sibling subjects

Twin and sibling participants (TSP) for the human study were recruited from southern California by access to a population birth record-based twin registry [15], as well as by newspaper advertisement [16]. The University of California San Diego, Institutional Review Board provided approval for the study and each subject or the parent of the minor subjects, gave written informed consent. A subset of 381 individuals of the TSP population was randomly selected and included 60 dizygotic (DZ) and 160 monozygotic (MZ) twin pairs. Zygosity of twins was confirmed genetically by use of microsatellite and single nucleotide polymorphism (SNP) markers [16]. Initially ethnicity was established by self-identification, including information on geographic origin of both parents and all four grandparents, and only individuals of Caucasian or Hispanic ancestry/ethnicity are included here. The age of the subjects ranged from 14 to 78 years, with a median of 39. Phenotyping (biochemical and physiological) was conducted as previously described [16]. All of the 381 TSP subjects with both genotypes and phenotypes were included in the analyses (see below).

### Molecular genetics, genomic DNA and genotyping

Genomic DNA was extracted from leukocytes in EDTA-anticoagulated blood after proteinase-K digestion of proteins, by adsorption/elution from Qiagen columns, as previously described [16], and genotyped for 592,312 SNPs using the Illumina 610-Quad genotyping array and passed final quality control (QC: see below). For each MZ twin pairs, only one individual underwent GWAS, and the genotype information was used for both members of MZ twins. During analysis, family structure was accounted for in MERLIN (see below).

### Biochemical assay of active renin in human plasma

EDTA–anticoagulated plasma samples were collected from seated subjects, and stored frozen at −70 **°**C until assayed. Circulating active renin was quantified at room temperature for 3 hours with a 2-site IRMA [17] wherein the mouse monoclonal anti human renin antibody was specific for a renin epitope formed after excision of active renin from pro-renin (DSL, Webster, TX; DSL-25100); the active renin assay sensitivity was ~0.48 pg/ml, with intra-assay coefficients of variation from 1.4-4.3 %, and inter-assay coefficients of 1.9-3.0 %.

### Genetic association analyses

To test SNP on phenotype effects with explicit accounting for family structure for the TSP cohort, MERLIN v1.1.2 (http://www.sph.umich.edu/csg/abecasis/merlin/) was used. As an additional QC step, unlikely genotypes based on expected inheritance patterns were removed using Merlin's Pedwipe procedure. A maximum likelihood estimation test of a variance components model was used, incorporating a variance-covariance matrix that allows for family relatedness, including twin status, to be modeled and appropriately controlled for in the association test. In addition, age, gender, and the first MDS component were included as covariates. A standard criterion of p < 5x10^−8^ across the genome was used to indicate significance of single SNPs on traits. The “Manhattan” plots visualized results across the genome, as well as local “SNAP” (SNP Annotation and Proxy Search) plots [18] <http://www.broadinstitute.org/mpg/snap/ldplot.php>.

### Replication Marine Resiliency Study (MRS)

We also measured active plasma renin (by ELISA) in samples from 799 healthy unrelated male Marines from the Marine Resiliency Study (MRS) with available genotypes [19]. The method for genotyping of MRS subjects has been detailed earlier [20, 21]. In brief, genotyping was carried out using the HumanOmniExpressExome (HOEE) array with 951,117 loci from Illumina (http://www.illumina.com/), resulting in a high initial locus success rate and overall data quality. Additional data cleaning was performed in PLINK v1.07 [22], using standard procedures. All subjects included here were active duty male and of European ancestry [23]. All subjects provided written consent for the genetic study. Association of plasma renin activity with genotypes were performed using a linear regression in PLINK (v.1.07) using age and 3 principal components (PC's) to correct for population stratification as covariates. We used the Genetic Power Calculator from Purcell et al. to estimate power [24]. Based on an effect size estimate of 1 % of variance explained by a candidate variant, we estimate that we had 83 % power to detect an effect of SNP on renin levels at an alpha level of 0.05, given the number of samples available in the MRS. Furthermore we estimate that we would have >94 % power to detect an effect of this size in a meta-analysis of the MRS and TSP.

### Meta-analyses

Results from the TSP and MRS data were combined in an inverse variance and weighted fixed-effect meta-analysis was carried out using METAL [25].

### Protein chemistry and enzymology

#### Digestion of recombinant human pro-renin by human KAL

Recombinant human pro-renin (5 μM) (Cayman Chemical, catalog number 10007599) was digested with protease human KAL (kallikrein, human plasma, Calbiochem, EMD Millipore, catalog number 420307, specific activity 15 U/mg protein) (1 μM) at 37 **°**C for 15 min in 12 μl of reaction volume with assay buffer (50 mM Tris, pH 7.5, NaCl 250 mM). The reaction was terminated by adding aprotinin (2 μM), purified by ZipTip (small C-18 column) and then analyzed by MALDI-TOF. For SDS-PAGE, pro-renin was incubated in absence or presence of KAL as mentioned above for 2 hours, and analyzed on 10 % or 4-12 % (gradient) NuPAGE gels.

#### Digestion of renin substrate angiotensinogen (AGT) with KAL-activated renin

Human pro-renin (5 μM) was digested with KAL (1 μM) in 50 mM Tris, pH 7.5 and NaCl 250 mM in a volume of 12 μl for 15 min at 37 **°**C, as mentioned above in the first step. In the second step, 12 μl of sodium acetate buffer, pH 5.5 containing angiotensinogen synthetic tetradecapeptide (14 amino acids; DRVYIHPFHL↓VIHN) (Phoenix Pharmaceuticals, Inc.) was added (in final concentrations of sodium acetate 0.2 M and tetradecapeptide 10 μM), and further incubated for another 15 min at 37 **°**C. The reaction digests were then purified through ZipTip adsorption/elution, and were analyzed by MALDI-TOF.

#### MALDI-TOF analysis

MALDI-TOF analyses were performed as described before using a PE Biosystems Voyager DeSTR MALDI-TOF mass spectrometer (Applied Biosystems, Foster City, CA) [26]. Resulting peptide masses were analyzed in the ProteinProspector Program (<http://prospector.ucsf.edu>) to identify the possible fragments of the respective proteins.

#### Identification of active renin and pro-renin protein bands in KAL digests, analysis by LC-MS/MS sequencing

Gel slices were cut, processed for in-gel trypsin digestion and the extracted peptides were analyzed by reverse-phase liquid chromatography (LC) in combination with tandem mass spectrometry using electrospray ionization with a QSTAR-Elite hybrid mass spectrometer (AB/MDS Sciex) as described before [27]. Peptide identifications were made using the Paragon algorithm executed in Protein Pilot 2.0 (Life Technologies).

#### Amino acid sequence analysis by TOF/TOF

Tandem mass analysis (MS/MS) for sequencing was performed on a 4800 MALDI-TOF-TOF mass spectrometer (Applied Biosystems) as described before [26].

### Mouse juxtaglomerular cell culture

Mouse kidney juxtaglomerular cells As4.1 (ATCC **®** CRL-2193™) were grown in DMEM high-glucose (GIBCO) with 10 % FBS and Penicillin/streptomycin/glutamine media at 37 **°**C with 5 % CO_2_.

### Co-localization of Renin and KAL by immunofluorescence

#### Mouse CRL-2193 (As4.1) juxtaglomerular cells

Cells were grown on cover slips, washed with PBS and were fixed with 2.5 % paraformaldehyde in PBS for 20 min at room temperature. Cells were then permeabilized with 0.5 % Triton in PBS for 10 min at room temperature. Cells were blocked using 5 % BSA in PBS for 30 min followed by primary antibody incubation [rabbit anti KAL (1:100, Bioss) and goat anti renin (1:100, Santa Cruz Biotechnology)] in 2 % BSA for 2 hr at room temperature. Coverslips were washed 3 times 5 min each and then incubated with secondary antibody Alexa Fluor 488 nm (green) coupled to donkey anti rabbit (1:250, Invitrogen) and Alexa Fluor 594 nm (red) donkey anti goat (1:350, Invitrogen) along with Hoechst 33342 (nuclear stain; 1 μg/mL) in 1 % BSA for 1 hr at room temperature. Coverslips were washed and mounted on glass slide using Slowfade-antifade (Molecular Probes). Images were acquired on a Delta Vision deconvolution microscope and SoftWorx software (Applied Precision, Issaquah, WA), using 60x objective as described previously [28].

#### Mouse kidney immunohistochemistry

Formaldehyde-fixed paraffin-embedded kidney tissue sections were cleared of paraffin and hydrated through graded alcohol and boiled in 100 °C for 20–30 min for antigen retrieval [29]. After permeabilization and blocking, sections were incubated overnight at 4 °C with primary antibodies to renin and KAL, followed by incubation with Alexa Fluor secondary antibodies as described above. Images were captured on a Delta Vision deconvolution microscope using 20x objective.

#### *REN* and *KLKB1* mRNA expression in organs and cells

Transcriptomes of mouse adrenal gland from mouse strains blood pressure high (BPH) and blood pressure low (BPL) (each in triplicate) [30]; rat adrenal gland (SHR and WKY strains, each in triplicate) [31] and mouse As4.1 juxtaglomerular cells (in duplicate) [32] were profiled by microarray analysis as previously described, and data are available at NCBI GEO. Data were globally normalized to median expression, and then analyzed statistically.

### Statistical analyses

The results were expressed as mean ± one SEM. Multiple comparisons were made using one-way ANOVA followed by Bonferroni post hoc tests, or by two-way ANOVA using Kaleidagraph (Synergy Software, Reading, PA). Statistical significance was concluded at *p* < 0.05.

## Results

### Meta-analysis of genetic association for polymorphisms at the *F12* and *KLKB1* loci and active renin concentration in plasma

The best-characterized functional polymorphism at the *KLKB1* locus rs3733402 results in loss-of-function amino acid substitution Asn124Ser [33]. This substitution in the apple 2 domain impairs binding and digestion of the classical substrate HMWK (high molecular weight kininogen) [14]. At the *F12* locus, the rs1801020 polymorphism is in the 5’-UTR (C46T) creates a new upstream translational start codon, thereby attenuating formation of the authentic F12 protease [34].

**Fig. 1:**
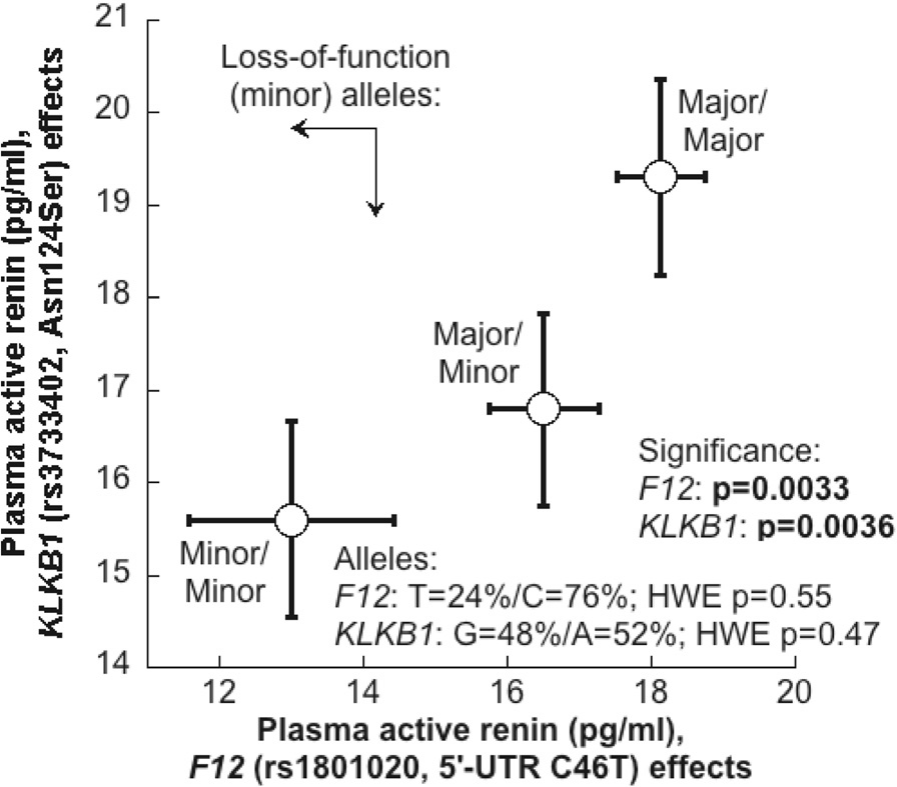
Effect of polymorphisms in the *KLKB1* (Asn124Ser) and *F12* (5’-UTR C46T) loci on circulating levels of active renin in UCSD twins/siblings and in MRS subjects. Polymorphisms at *F12* and *KLKB1* loci influenced plasma active renin levels in two independent cohorts civilian and marines. Each SNP was in Hardy-Weinberg equilibrium with *p* > 0.05

Since these proteases are part of the kallikrein-kinin system and interact with each other at the molecular level, we looked at genetic association of the polymorphisms described above with levels of active renin in plasma. The effect of the human polymorphisms rs3733402 in the *KLKB1* locus and the rs1801020 in *F12* locus were very significant on the active renin levels in plasma of both the TSP and MRS populations (Table 1, Fig. 1). In both cases, minor alleles were associated with low levels of active renin in the plasma (Fig. 1). Meta-analysis combining the TSP and one independent population (MRS) for a total of *n* = 1,180 subjects, indicated allelic effects consistent in magnitude (beta, or effect size per allele) and direction (sign on slope) across populations. The overall slope of the meta-analysis regression for rs3733402 and rs1801020 was beta = 0.055 and 0.057, with SE =0.014 (p = 6.83 x 10^−5^) and = 0.016 (*p* = 0.0003) respectively (Table 1).

**Table 1:** Meta-analysis of the effect of *KLKB1* and *F12* genetic polymorphisms on generation of active renin in human plasma

*KLKB1* (rs3733402)														
Cohort	A1	A2	N	BETA	SE	P	MAF	HetISq	HetP	G/G Freq	G/A Freq	A/A Freq	HWE chi-square	HWE-p
TSP	G	A	381	−0.071	0.025	0.005	0.5			20.42%	49.17%	30.42%	0.399	0.712
MRS	G	A	799	−0.048	0.016	0.0037	0.47			20.40%	52.82%	26.78%	2.67	0.102
Meta-analysis	G	A	1180	−0.055	0.014	7.22E-05	0.48	0	0.507					
*F12* (rs1801020)														
Cohort	A1	A2	N	BETA	SE	P	MAF	HetISq	HetP	A/A Freq	A/G Freq	G/G Freq	HWE chi-square	HWE-p
TSP	A	G	381	−0.061	0.031	0.0459	0.23			4.58%	33.75%	61.67%	0.514	0.426
MRS	A	G	798	−0.055	0.018	0.0026	0.24			6.14%	35.71%	58.15%	0.339	0.0561
Meta-analysis	A	G	1179	−0.057	0.016	0.0003	0.24	0	0.947					

A1/A2: effect allele/non-effect allele, N: sample size, BETA: estimated beta coefficient, SE: standard error of beta, P: p-value for beta, MAF: minor allele frequency, HetP: p-value for Cochran's Q statistic, HetISq: I^2^ heterogeneity index, TSP: twin & sibling participants, MRS: Marine resiliency study

### Digestion of human recombinant pro-renin with kallikrein (KAL) yields active renin and the pro-peptide byproduct

**Fig. 2:**
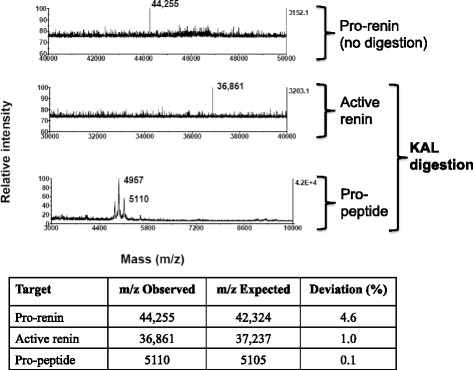
Mass spectrometric analysis of the KAL digested samples of recombinant pro-renin. Recombinant pro-renin was incubated in absence (upper panel) or presence (middle and bottom panel) of KAL in the assay buffer as mentioned before. The digestion mixture was acidified and purified through ZipTip and subjected to MALDI-TOF analysis in linear mode. Observed masses were compared with the theoretical mass predicted by ProteinProspector program and are shown in the table

**Fig. 3:**
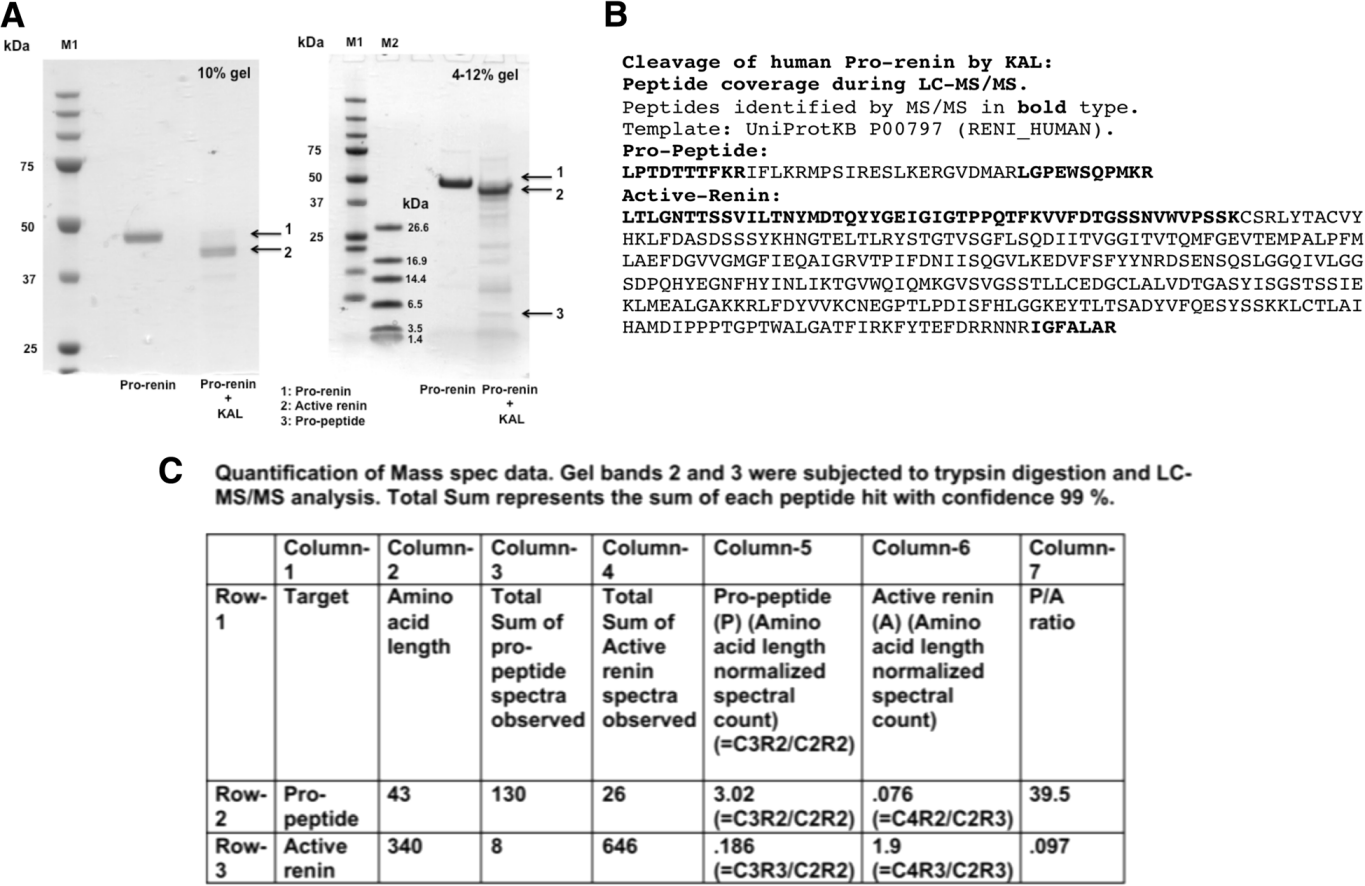
Molecular mass analysis of fragments generated by KAL digestion of pro-renin; identification of active renin and pro-peptide fragments. **a** Recombinant pro-renin was digested with KAL as described in Fig. 2 and subjected to SDS-PAGE; 10 % gel (left panel) for separation of active renin from pro-renin and 4–12 % gel (right panel) for identification of lower (~5 kDa) fragment). Fragments were numbered as 1 (pro-renin), 2 (active renin) and 3 (pro-peptide). M1 and M2 are SDS-PAGE molecular weight standards in broad range and low range respectively **b** Fragment 2 and 3 were cut out and subjected to trypsin digestion and LC-MS/MS analysis separately for identification. Peptide coverage (bold type) identified by MS/MS in the termini of pro-peptide (from fragment 3) and active renin (from fragment 2) is shown. **c** Quantification of the MS data. The total sum of pro-peptide and active renin spectra observed were normalized by the amino acid length (43 for pro-peptide and 340 for the active renin) to represent the pro-peptide to active renin ratio in gel fragments 2 or 3

MALDI-TOF analysis of KAL digested pro-renin displayed two peaks of m/z 36,861 and 5100, corresponding to the theoretical masses of active renin and pro-peptide respectively (Fig. 2, lower panels). In control reaction, where pro-renin was incubated in absence of KAL, MS chromatogram showed a single peak of m/z 44,255, representing the intact pro-renin (Fig. 2, upper panel). In order to identify the sequence of the digested products, the digestion mixture was subjected to SDS-PAGE on a 10 % gel to separate high molecular weight pro-renin and active renin, and on a 4-12 % gradient gel to separate low molecular weight pro-peptide fragment. A faster migrating band compared to that of pro-renin appeared only in the KAL digested sample (Fig. 3a, marked with arrow 2). Generation of a low molecular weight fragment of ~ 5 kDa was evidenced after digestion of pro-renin with KAL (Fig. 3a, right panel, marked with arrow 3). Fragments marked with arrow 2 and 3 were cut out from the gel, trypsin digested and subjected to LC-MS analysis for identification. Peptides identified from gel fragment 3 showed significant coverage on the N and C-terminal of pro-peptide sequence (Fig. 3b), whereas same from gel fragment 2 showed coverage on active-renin (Fig. 3b). Since LC-MS analysis from gel fragment 3 identified some active renin sequence and gel fragment 2 identified some pro-peptide sequence, we quantified the data by normalizing the total sum of spectra for pro-peptide and active renin observed in gel fragment 2 and 3 by their amino acid length. Quantification of mass spec data showed a significant enrichment (400-fold) of pro-peptide to active renin ratio in gel fragment 3 over gel fragment 2 (Fig. 3c).

### KAL digested pro-renin cleaves angiotensinogen substrate to generate angiotensin I

**Fig. 4:**
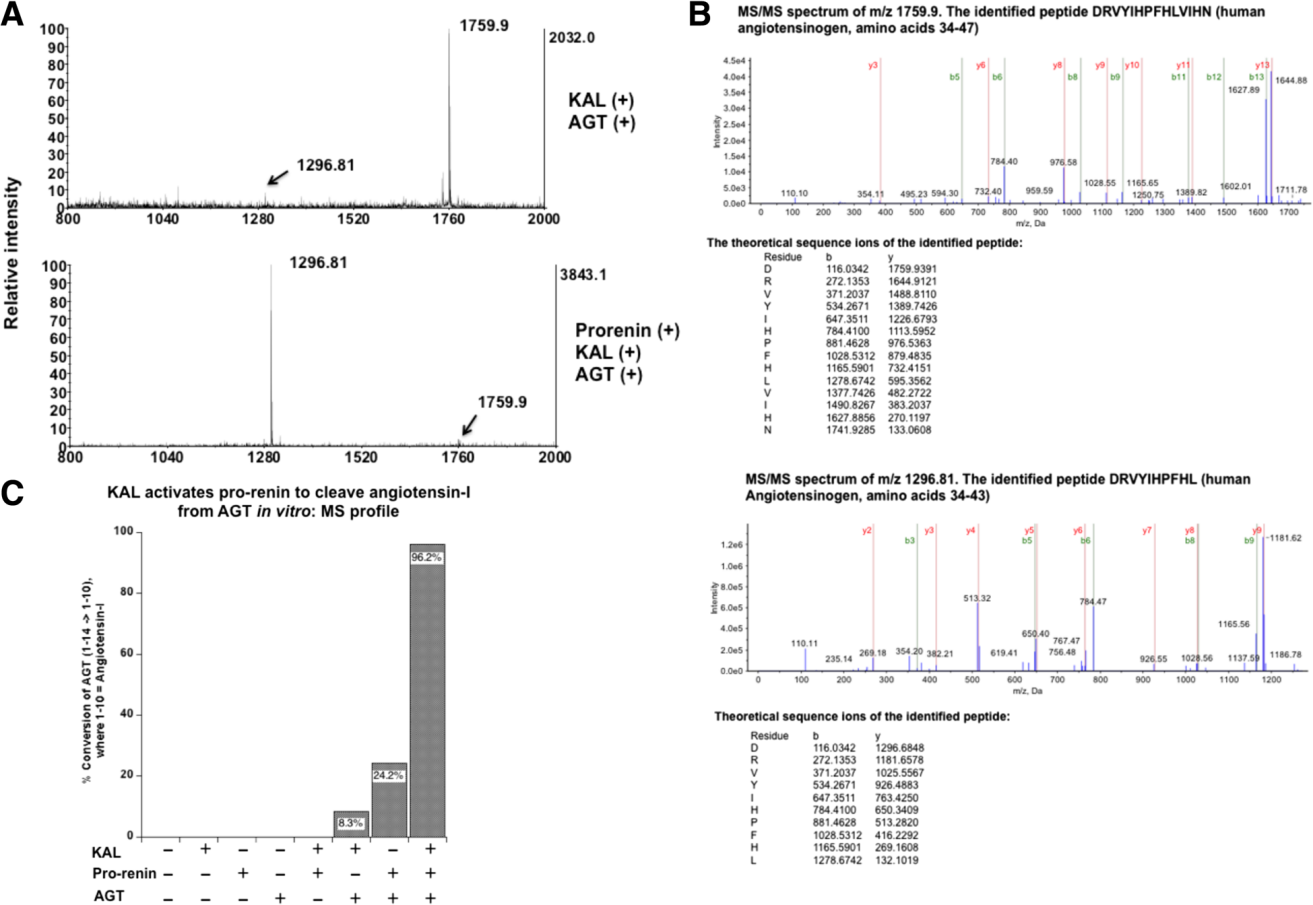
Generation of Ang I from renin substrate tetradecapeptide (pre-angiotensinogen 1–14) by KAL-activated pro-renin.** a** AGT was incubated with KAL (upper panel) and with pro-renin and KAL (lower panel) as described in methods. The reaction digests were purified through ZipTip and subjected to MALDI-TOF analysis. **b** TOF/TOF analysis of the precursor mass 1759.9 and 1296.81 **c** Quantification of the MS scans. The % conversion of AGT (1–14 to 1–10) was quantified from the relative intensity of the Ang I peptide under different experimental conditions

Active renin digests substrate angiotensinogen to generate angiotensin I (Ang I). We tested the ability of KAL digested pro-renin to digest angiotensinogen. The pre-angiotensinogen 1–14 tetra deca peptide (AGT) was incubated with the KAL-digested pro-renin. Analysis of the digestion reaction containing AGT and KAL revealed one major peptide of m/z 1759.9 (Fig. 4a, upper panel). Incubation of pro-renin with KAL followed by the addition of AGT generated a major peak of m/z 1296.81 (Fig. 4a, lower panel). MS/MS analysis of the precursor mass 1759.9 and 1296.8 confirmed the sequence of these two peptides as amino acids 34–47 and 34–43 of human angiotensinogen (Fig. 4b). Quantification of MS data suggest ~ 96 % generation of Ang I peptide in reaction containing KAL, pro-renin and AGT, whereas only 24 % in presence of pro-renin and AGT and 8 % in presence of KAL and AGT. The generation of Ang I or AGT 1–10 peptide of m/z 1296.8 was not detected in digestion reactions containing only KAL, pro-renin, AGT or in KAL and pro-renin combination (Fig. 4c).

### Renin co-localized with KAL, in kidney JG cells and their renin secretory granules

**Fig. 5:**
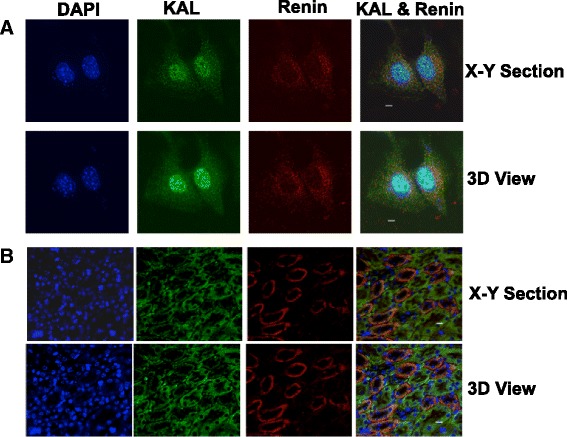
Co-localization of renin and KAL in **a** As4.1 (CRL-2193) juxtaglomerular cells and in **b** mouse kidney: The cells (upper two panels) and kidney sections (lower two panels) were stained with rabbit anti-KAL primary and donkey anti-rabbit IgG- Alexa Fluor®488 conjugated secondary (green); and goat anti-renin primary and donkey anti-goat IgG- Fluor®594 conjugated secondary (red). The nuclei were visualized with Hoechst 33342 dye (blue). A series of *xy* optical sections along the z-axis was acquired using a 60x oil immersion objective for cells and 20x objective for kidney section by deconvolution microscopy and the data set was processed to generate three-dimensional (3D) or representative *xy* section. Co-localization of KAL (green) and renin (red) is shown by overlay of the images (yellow fluorescence). Bar scale is 5 μM

Immunofluorescence experiments of mouse juxtaglomerular cells (Fig. 5a) as well as in mouse kidney section (Fig. 5b), was used to establish renin’s subcellular co-localization with its processing enzyme KAL. The immunofluorescence micrographs showed that renin and KAL co-localized partially as evidenced by the orange/yellow fluorescence in the overlay figures. Pearson coefficient of co-localization was 0.15 for As1.4 cells and 0.5 for the kidney section.

### Endogenous expression of *KLKB1* and *REN*

**Fig. 6:**
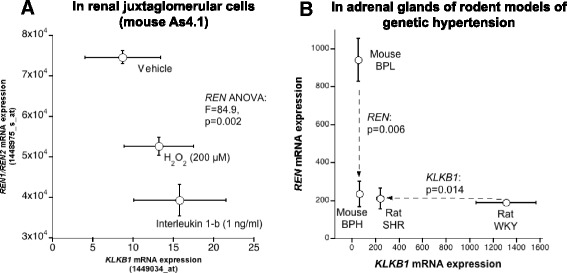
Correlation of *REN* and *KLKB1* mRNA expression in **a** renal juxtaglomerular cells and (B) in the adrenal glands of rodent models of genetic hypertension (mouse: BPH/BPL and rat: SHR/WKY): *REN* and *KLKB1* mRNA expression data were collected and compared for (**a** and **b**)

After confirming by in vitro assay that KAL processed prorenin to active renin, we analyzed how the expressions of *KLKB1* and *REN* genes might be correlated under various physiological conditions. *REN* and *KLKB1* mRNA expression data were collected and analyzed for in mouse As4.1 cells (Fig. 6a) and adrenal tissues of rodent genetic hypertension models: blood pressure low (BPL) and blood pressure high (BPH) mouse models and normotensive Wistar-Kyoto (WKY) and spontaneously hypertensive (SHR) rat models (Fig. 6b). In As4.1 cells feedback inhibition of renin expression was observed by the addition of interleukin 1-β or hydrogen peroxide, concomitantly KLKB1 expression remained unaltered. The hypotensive phenotype of BPL mice triggered renin expression, ~ 4 fold higher compared to hypertensive BPH mice. However the expression of KLKB1 did not differ significantly amongst BPL and BPH mice. The normotensive WKY rats have significantly higher *KLKB1* expression (~5.5 fold) compared to the hypertensive SHR rats with more or less similar level of REN mRNA expression in both rat models. Thus regulation of blood pressure under various physiological conditions may involve modulation in the expression of either *REN* or *KLKB1*.

## Discussion

In vitro studies have demonstrated that proteases such as trypsin, plasmin, pepsin, kallikrein and several others activate zymogen pro-renin to active renin [35–38]. Studies before the era of mass spectroscopy suggested involvement of KLKB1 and FXIIa in pro-renin processing [39–41]. Genetic variation at the *KLKB1* locus (encoding for plasma pre-kallikrein or Fletcher factor; EC 3.4.21.34) was previously most widely investigated for its roles in coagulation and allergy. We demonstrate using in vitro enzymatic assay the ability of active protease KAL in processing pro-renin → renin. A second association of renin activity and the protease *F12* locus (encoding for Factor XII or Hageman factor; EC 3.4.21.38) suggests a cascade of enzymatic events (FXIIa → KAL) in control of pro-renin activation. Generation of active renin by the cascade thus provides evidence of a site for BP regulation.

The *KLKB1* locus lies directly beneath a previously described LOD peak (LOD = 3.2) for BP on chromosome 8 in the genetically hypertensive strain of mice (BPH) [42]. We therefore explored the effects of *KLKB1* genetic variation upon formation of active renin. While most of the *KLKB1* single nucleotide polymorphisms (SNPs) reported are located in the non-coding regions, rs3733402 in exon 5 results in an amino acid substitution Asn124Ser [14, 33]. This mutation in the apple domain 2 of heavy chain reduces the binding of KAL to its substrate HMWK, and therefore this SNP was chosen to investigate its association with prorenin processing. Indeed, an immunoassay specific for active renin revealed that Ser/Ser homozygotes had lower circulating active renin (Fig. 1), consistent with diminished pro-renin cleavage by a less active Ser allele. Previously, rs3733402 has shown strong association with pre-pro-endothelin-1 and pre pro-adrenomedullin in the Prevention of Renal and Vascular End stage disease (PREVEND) study [43]. In the recent study by Lieb et al. the top SNPs identified were rs12374220, an intronic variant in the *TENM3* gene, rs5030062 in the intron 6 of kininogen 1 gene and rs4253311 in intron 11 of the kallikrein B (*KLKB1*) gene. The intronic SNP rs4253311 provided no evidence for association with renin concentrations and explained 0.87 % of plasma renin activity variance [44]. In our study MALDI mass spectrometry documented the formation of active renin and the pro-peptide after digestion of pro-renin with KAL (Fig. 2 & Fig. 3). Furthermore the sub-cellular co-localization of renin with KAL suggests molecular interaction between these two proteins (Fig. 5a &b). Renin immunoreactivity has previously been shown in the cytoplasmic granules of cultured JG cells and in kidney sections [45]. The cleavage sites involved in pro-renin processing include lysine-arginine, which is the recognition site of plasma kallikrein [46]. Our genetic and biochemical data suggests an enzyme-substrate relation between KAL and prorenin. This suggests the possible existence of feedback regulation at the molecular level in the events leading to active renin generation by KAL and BP regulation.

KAL is a glycoprotein that takes part in the surface dependent activation of blood coagulation, fibrinolysis and kinin generation. It is synthesized in the liver and secreted into the blood as prekallikrein, which is then converted to active plasma kallikrein by factor FXIIa [47]. The C46T 5’-UTR polymorphism associated with Hageman factor has been described to be associated with its plasma concentration and thrombotic risk [48, 49]. The KAL protease might catalyzes the conversion of HMWK to bradykinin in one hand, and the active renin on other hand. The downstream target angiotensin converting enzyme (ACE) then modulates the concentration of angiotensin II, the key player of the RAAS system, and bradykinin, a component of the kallikrein-kinin system in opposite direction, therefore establishing a direct interaction between kallikrein-kinin and renin-angiotensin system [50, 51].

The genetic variation in the *F12* and *KLKB1* loci directly affecting their amino acid sequence could ultimately influenced the processing, secretion or circulation of the active renin protein, which in turn mediates the BP phenotype. Allelic effects might also act on the cluster of characteristics associated with cardiovascular risk for which plasma renin is a biomarker. In the coagulation system, it has been reported that even the homozygous deficiency of the *KLKB1* loci results in no discernible coagulopathy [52]. In treatment of hereditary angioedema inhibition of KAL does play a beneficial role, perhaps by inhibition of bradykinin formation [53].

### Advantages and limitations

Here we report a comprehensive GWAS showing correlation between polymorphisms at two independent loci (*KLKB1* rs3733402 and *F12* rs1801020) and plasma renin activity. Cellular and biochemical evidence is provided to establish that correlation. To our knowledge this is the first report of SNPs in two independent loci with significant trait association with activation of renin-angiotensin system. This study focused on the best characterized SNP (rs3733402) in the exon 5 of *KLKB1* gene. Although association of kallikrein with renin activation has previously been described, adequate information on direct in vitro protease biochemistry was lacking. Therefore we used a mass spectrometry approach to characterize in vitro digestion of prorenin by KAL to reestablish kallikrein association with prorenin processing. In the scenario of this genetic association, the efficacy of digestion of prorenin by mutant KAL (Asp124Ser) needs to be compared with that of the wild type KAL. We have not addressed in these populations the active plasma renin association with the previously described intronic variant at *KLKB1* (rs4253311) and other SNPs. Future studies will explore the association of these two SNPs with BP, renal and/or metabolic traits.

## Conclusion

**Fig. 7:**
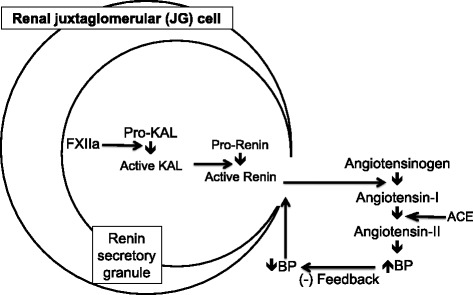
Hypothetical schematic representing activation of pro-renin by a proteolytic enzyme cascade of FXIIa → KAL, with consequences for regulation of BP. Pro-renin processing within the secretory granule of renal juxtaglomerular cells by sequential enzymatic events catalyzed by F12 and KAL

Our findings draw attention to the role of KAL as a pro-renin convertase and suggest a potential target for inhibition of the rate-limiting step in the RAS pathway. Polymorphisms at the *KLKB1* (rs3733402) and *F12* (rs1801020) loci are associated with low active plasma renin activity. Genetic, cell and biochemical studies suggest a cascade of enzymatic events involving factor FXIIa activation of prekallikrein to active kallikrein in control of pro-renin activation. Thus plasma kallikrein presents potential as novel therapeutic target for blood pressure regulation with implications of KAL inhibition for treatment of hypertension (Fig. 7).

## Abbreviations

AGT: Angiotensinogen
Ang I: Angiotensin I
Ang II: Angiotensin II
BP: Blood pressure
Factor XII (FXII Hageman factor): protein encoded by gene *F12*
JG: Juxtaglomerular
KAL: Kallikrein
MAF: Minor allele frequency
MALDI-TOF: Matrix Assisted Laser Desorption Ionization/Time Of Flight.
Prekallikrein (prokallikrein Fletcher factor): protein encoded by gene *KLKB1*
RAAS: Renin-angiotensin-aldosterone system
SNPs: Single nucleotide polymorphisms
TSP: Twin and sibling participants
